# Differential Background Music as Attentional Resources Interacting with Cognitive Control

**DOI:** 10.3390/ijerph192215094

**Published:** 2022-11-16

**Authors:** Ga Eul Yoo, Sujin Lee, Aimee Jeehae Kim, Seung Hong Choi, Hyun Ju Chong, Sunghyouk Park

**Affiliations:** 1 Department of Music Therapy, Graduate School, Ewha Womans University, Seoul 03760, Republic of Korea; 2 Natural Product Research Institute, College of Pharmacy, Seoul National University, Seoul 08826, Republic of Korea; 3 Department of Musicology and Culture, Music Therapy Major, Graduate School, Dong-A University, Busan 49315, Republic of Korea; 4 Department of Radiology, Seoul National University College of Medicine, Seoul 03080, Republic of Korea

**Keywords:** background music, cognitive performance, attentional control, music majors

## Abstract

We examined the effects of background music on cognitive task performances using different musical arrangements from an excerpt of Mozart’s Piano Sonata K.448. The participants were 126 university students: 70 music majors and 56 nonmusic majors. Three types of musical arrangements were used as background conditions: rhythm-only, melody, and original music conditions. Participants were asked to perform cognitive tasks in the presence of each music condition. The participants’ percentage of completed items and accuracy on these tasks were compared for music and nonmusic majors, controlling for the effect of perceived level of arousal and their performance during no background music. Whether a participant’s perceptions of background music predicted their cognitive performance was also analyzed. We found that music majors demonstrated decreased task performance for the original background condition, while nonmusic majors demonstrated no significant differences in performance across the arrangements. When pitch or rhythm information was modified, emotional valence and arousal were perceived differently. Perception of the complexity of the background music depending on the arrangement type differed between music majors and nonmusic majors. While the perceived complexity significantly predicted nonmusic majors’ cognitive performance, its predictive effect was not found in music majors. The findings imply that perceptions of musical arrangements in terms of expectancy and complexity can be critical factors in determining how arrangements affect concurrent cognitive activity, while suggesting that music itself is not a facilitating or detrimental factor for cognitive performance.

## 1. Introduction

The effect of background music on cognitive performance has received considerable attention over the last few decades [1,2]. However, the impact of background music in terms of facilitating or interfering with concurrent cognitive tasks remains inconclusive. While some studies support that background music enhances cognitive performance, other studies have found that background music is either detrimental to or has no impact on such performance [1,3]. One of the reasons music is presumed to improve cognitive performance is related to the finding that music listening increases positive emotions (or pleasantness) and arousal [4,5]. Multiple studies found favorable changes in behavioral measures of attention in the presence of background music compared to silence or other types of auditory background noise [6,7,8,9]. For example, in one study, music was found to lead to an optimal level of arousal, which facilitated the use of the effective selective attention strategy [10]. Neurological evidence of such changes includes brain networks intervening in attentional control and inhibitory cognitive processing being recruited by music that is perceived as highly arousing or joyful [11,12], which supports the idea that music helps individuals focus on target stimuli and ignore competing stimuli. These studies commonly highlight that the emotional valence (i.e., positive or negative) and arousal state induced by music mediate attentional processing.

However, the findings from other studies question the beneficial effects of music on task performance. For example, it was found that the silent condition led to better performance or no differences compared to the music conditions [13,14]. Another study found that highly arousing music resulted in poorer task performance [15]. The researchers suggested that music that was perceived as too stimulating and arousing might interfere with cognitive processing. It was also reported that preferred music or familiar music could disrupt cognitive task performance by diverting attention away from the target task [16,17,18,19]. These results imply that background music demands cognitive processing, because it contains elements (e.g., a melodic or chord progression) that attract listeners’ attention. Accordingly, music, as a competing stimulus, draws attention away from the target task [18] and interferes with the process of inhibiting unnecessary processing.

Such contradictory findings align with the ongoing debate on the so-called Mozart effect. Early studies supporting that listening to Mozart’s music improved cognitive task performance (e.g., spatial processing) led to the term “Mozart effect” and its overemphasis on the beneficial effect of the music by a specific composer. Subsequent studies have replicated the effect while others have disproved it with some meta-analyses demonstrating inconsistent results [1,3,20].

The effect of background music on cognitive performance has also been discussed in terms of individual differences. In particular, research has identified personality (e.g., introvert vs. extrovert) as factors that affect information processing [21]. Those studies found that introverts and extroverts showed a different level of cortical arousal at rest; accordingly, background music is either necessary or unnecessary to stimulate their arousal to an optimal level. This phenomenon is explained by Eysenck’s theory of personality [21,22,23]. Those who present conflicting findings against this theory instead suggest that the key for improved cognitive performance is the arousal level induced prior to cognitive performance through music listening, regardless of the task performer’s personality [21].

These findings indicate that the mere presence or absence of background music does not determine whether and how music influences cognitive performance. Attentional control theory identifies attention as a critical factor in cognitive performance and proposes that successful attentional control (i.e., selecting the relevant information to be focused on and filtering out the competing or irrelevant information) is mediated by multiple factors and the interactions between them [24,25]; it also emphasized the individual differences in such attentional control being enhanced or interfered with [24]. This theoretical framework calls for the systematic investigation of the interactions between type of stimuli as an information source and individual characteristics, which could explain the underlying mechanism resulting in contradictory findings regarding the role of background music on cognitive performance. Nonetheless, there have been few empirical studies using this framework.

Some studies have begun to investigate how individual factors affect the effect of music in cognitive performance. In those studies, intramusical elements have been investigated in relation to individual attributes. In a study that compared different modes of music, female participants performed significantly better on verbal tasks with major modes [26,27]. Another study demonstrated poorer performance in language comprehension and visuospatial tasks in the presence of background music for musicians compared to non-musicians [28], which suggests that music training can impact how individuals process background music while concurrently engaging in cognitive tasks. Another interesting study demonstrated that musicians tended to be distracted by music played by their primary instruments [29] These findings indicate that individuals would shift their attention away from a concurrently presented cognitive task depending on how much they apply their resources to process music as a competing stimulus.

Along with this stream of research, this current study considers the interactions between intramusical factors (specific attributes of the music that determine its relevance to the task) and individual factors (music-related background of task performers and their perceptions of the music, which could affect their level of arousal based on the perceived predictability of music) as an underlying mechanism of how specific background music can potentially facilitate or hinder task performance. We based our hypothesis on the theory that how much the listener consciously segregates or integrates streams of sounds determines their attentional load, which must be focused on the target stimulus to effectively complete the cognitive task [30,31]. Moreover, this process differs across individuals based on what types of resources, associations, and capacity they have, which affect the level of arousal and attentional processing [32]. Accordingly, we adjusted the combinations of musical elements by extracting or modifying a certain element (i.e., rhythm or melodic component) to see how such musical construction affects information load and whether such influence varied between music majors and nonmusic majors who had different perceptual musical schemes based upon their past musical experience and training.

Therefore, in this study, we aimed to examine the effects of background music using different musical arrangements with distinct information (i.e., rhythm, melody, or combination of multiple musical elements) and compared the effects of these arrangements on cognitive performance (i.e., simple visuo-spatial task) between music and nonmusic majors. Furthermore, we investigated how individual perceptions of the music (i.e., complexity and perceived emotional arousal and valence in the presented musical arrangement) and task performance were inter-correlated for music majors and nonmusic majors.

## 2. Materials and Methods

### 2.1. Participants

All procedures related to this study were approved by the Institutional Review Board of Ewha Womans University (IRB No. 157-13) and carried out in accordance with the relevant ethical guidelines and regulations. Participants were undergraduate and graduate students. Flyers describing the study’s purpose, the research procedures and the participants’ rights were posted in universities located in four different provinces in Korea. Each participant voluntarily agreed to participate in this study. After signing the written consent form, they participated in listening experiments in a quiet and independent place at their university. In addition to their current university major (music versus nonmusic major), the duration of the participants’ private music-related training (i.e., private lessons excluding public music classes at school) was considered. For nonmusic majors, although they were not currently majoring in music, they were excluded from the study if they had received over 3 years of music training. Initially, 169 students participated in this study, but 126 were included in the final analysis after incomplete responses to the questionnaire were excluded: 70 music majors and 56 nonmusic majors who had not received such training. The average age for the music majors was 23.0 years and for nonmusic majors it was 22.0 years. On average, the nonmusic major group had 0.9 years of music training, and the music major group had 10.9 years of training, which was a significantly longer duration of music training. With regard to listening habits, the average number of hours spent listening to music did not significantly differ between the groups. Although the reported frequency of listening to music while performing other tasks was similar in the two groups, listening to music while not performing other tasks was significantly more frequent among music majors than nonmusic majors. Participants’ demographic information is displayed in Table 1.

### 2.2. Musical Excerpts

In this study, we extracted musical elements from an original musical arrangement to adjust musical information. Mozart’s Piano Sonata K.448 was used for the different musical arrangements. This music has been repeatedly used to examine its effect on cognitive performance [1,8,10,19]. This music is characterized as having structural regularity and clear and repetitive musical motives. The beginning part of the music was recorded and edited using the Musical Instrument Digital Interface in three arrangements: rhythm-only, melody-only, and original versions. Given that the focus of this study was to examine the differential effect of musical elements, the musical stimuli were constructed to minimize the features of other intramusical elements in the melody-only and rhythm-only versions: using isochronous beats for the melody-only version and using non-pitched instruments for the rhythm-only version. For the melody-only and original arrangements, the timbre of the piano was used; a non-pitched percussive instrument (i.e., drum) was used for the rhythm-only version. Details about each arrangement are summarized in Table 2.

### 2.3. Measures

#### 2.3.1. Perceived State of Arousal Scale

Given that the state of being alert is an influencing factor for any cognitive task, participants completed the Perceived State of Arousal Scale [9,18] before engaging in any other tasks. For this measurement, nine items related to arousal (i.e., alert, arousing, fatigued, inactive, powerful, quiet, sleepy, slow, and worn out) were presented and the participants were asked to rate the extent to which the presented arousal-related word indicated how they felt at the present moment. A 5-point rating scale was used, and participants wrote down the number corresponding to their rating from 1 (very slightly or not at all) to 5 (extremely) next to the word. Accordingly, the level of perceived alertness could range from 9 (the least alert) to 45 (the most alert).

#### 2.3.2. Frankfurter Attention Inventory Test

To investigate the role of background music in influencing attentional focus and allocating attentional resources to process multiple stimuli, we selected a cognitive task that required not only the detection of the target stimuli, but also inhibitory response to ignore similar but task-irrelevant stimuli. The Frankfurter Attention Inventory (FAIR) measures sustained attention, particularly the mental ability to attend to external stimuli and discriminate a target among stimuli with high similarity [33]. During FAIR administration, participants are asked to mark a target (circles with three dots and squares with two dots) among similar patterned test items by drawing a continuous line below the items and making a spike into the target within a presented time period.

#### 2.3.3. Perceptions of Background Music

After completing the cognitive task, participants were asked how they perceived the presented background music. Specifically, participants were asked to rate whether the background music was interfering in (−5) or facilitating (5) their performance. Moreover, they rated how they perceived the complexity, level of arousal, and valence of the music. An 11-point rating scale was used with opposite adjectives at the ends of the scale (i.e., simple to complex, negative to positive, and relaxing to arousing), and the directionality and intensity of the perceived responses were measured within the range of −5 to 5.

### 2.4. Procedures

Prior to the presentation of the cognitive task, participants completed a questionnaire on their music training and listening habits. In particular, on a 5-point Likert scale, they rated how often they listened to music while performing other tasks. Then, they rated how alert they were feeling while participating in this study. For the cognitive task (i.e., the FAIR test), participants performed the task for 90 s without music playing in the background and then they did it again with the three different arrangements, each one lasting for 90 s. The order of the background music was counterbalanced by using computer-generated random numbers. Musical stimuli were delivered through a speaker (SoundLink Revolve, Bose Corporation, Framingham, MA, USA) with loudness controlled within the range of 70–80 dB and optimal loudness adjusted for engaging participants in each experimental trial. After completing each cognitive task, participants also rated how they perceived the music presented while they were performing the task in terms of complexity, arousal of emotion, and valence of emotion. Once their participation in the study was completed, a $5 gift card was given to each participant as compensation for their participation.

### 2.5. Data Analysis

The data were analyzed using SPSS 25.0 (IBM, Armonk, NY, USA). For task performance on the FAIR, two measures were obtained: the percentage of total items completed and the percentage of correctly marked items. The percentage of completed items indicates the processing speed and the percentage of correct items indicates the accuracy of task performance. Statistical analyses were implemented for these two measures separately.

First, to see how background music, in terms of arrangement type, affected cognitive performance, the relationship between the ratings on whether the background music interfered with (or benefitted) task performance and the arrangement type were analyzed using a chi-square test. Moreover, to compare actual cognitive performance measures depending on the arrangement (i.e., rhythm-only, melody-only, and original versions) across groups (i.e., music majors and nonmusic majors), we used a mixed model of repeated measures ANOVA with the within-subject factor of arrangement type and between-subject factor of the major. We also controlled for the effect of the perceived level of arousal and level of performance during silence (no background music condition), while using the measures as covariates. In terms of perception of the music presented (e.g., perceived arousal, valence, and complexity) while performing the cognitive task, we carried out a multivariate ANOVA. Furthermore, we implemented multiple regression to investigate whether the perception of the music predicted cognitive performance (i.e., percentage of completed items and correctly performed tasks). These analyses were conducted for each group and compared between groups.

## 3. Results

The participants were 126 university students. Since the level of arousal can influence cognitive task performance, and the varied cognitive task performance at baseline may influence the effects of background music on concurrent cognitive performance, we confirmed the homogeneity of the groups (i.e., music majors and nonmusic majors) in terms of these variables. An independent *t*-test demonstrated that the music majors and nonmusic majors were not significantly different in their level of perceived arousal or cognitive task performance when there was no background music. The results are displayed in Table 3.

Participants were asked to rate on an 11-point Likert scale (with rating from −5 to −1 being detrimental, 0 being neutral, and 1 to 5 being beneficial) whether the presented background music was detrimental, neutral, or beneficial for their performance of the task, and the results are shown in Table 4. With regard to the interference or benefits of background music, each arrangement type was rated differently (**χ^2^** = 29.412, *p* < 0.001). While over 70% of participants perceived the melody-only version as interfering with their cognitive performance, for the original version, less than 50% perceived it as interfering and around 40% perceived the music as beneficial.

**Figure 1:**
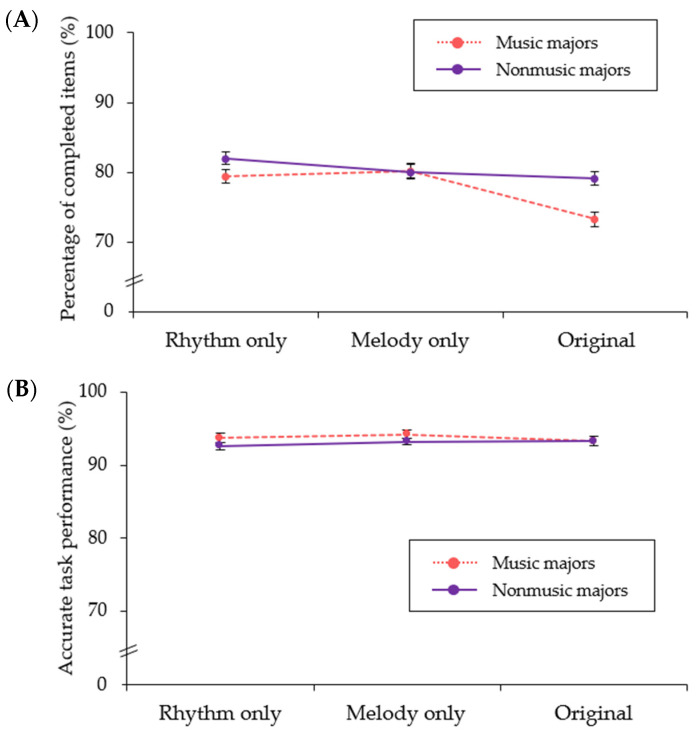
Percentage of completed items (Panel (**A**)) and accurately marked items (Panel (**B**)) on the FAIR by musical arrangement and group. Error bars indicate standard errors.

The participants’ cognitive performance results are displayed in Table 5. We conducted a mixed model of repeated measures ANOVA to compare cognitive performance depending on different arrangements of the background music, while controlling for the level of perceived arousal and performance when there was silence (i.e., baseline data for the task performance; see Table 6). In terms of percentage of completed items, the main effects of musical arrangement type and group were not significant, indicating the estimated means of completed items were not significantly different among the arrangement types or between the groups. Meanwhile, the interaction effect between arrangement type and group was significant. Nonmusic majors tended to complete a similar level of task items across the arrangement types, while music majors completed a similar rate of items for the melody-only and rhythm-only versions but a lower rate of items for the original condition (see Figure 1). In terms of correct task performance, the main effect of arrangement type was significant, although the post hoc analysis did not demonstrate significant differences among the paired comparisons of arrangement types. Meanwhile, the main effect of group and the interaction effect between the arrangement type and group were not significant.

We also examined how the music majors and nonmusic majors perceived the presented background music in terms of its complexity, and level of emotional arousal and valence while concurrently performing a cognitive task. The mean rating on music in each arrangement type is summarized in Table 5. We conducted a two-way MANOVA to examine the combined differences of the three measures of perception. The results (see Table 7) demonstrated that main effects of arrangement and group were statistically significant, indicating that the arrangement type and group (i.e., music majors versus nonmusic majors) were associated with different perceptions of the musical arrangements. Further analyses of between-subject effects (see Table 8) demonstrated which single measure (i.e., complexity, valence, or arousal) contributed to the significant difference. With regard to arrangement type, significant differences were observed in all three measures.

**Figure 2:**
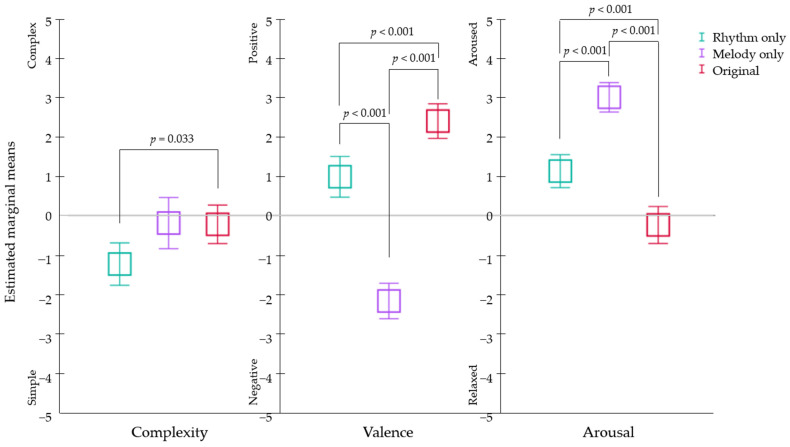
A between-group comparisons of perception of background music during cognitive task performance. Error bars represent 95% confidence intervals.

Post hoc analyses with paired comparisons for each measure demonstrated that a significant difference in perceived complexity was found between the rhythm-only and original version (*p* = 0.033), indicating that participants perceived the original version as being significantly more complex than the rhythm-only version. Other comparisons did not reach statistical significance. For perceived valence and arousal, all paired comparisons reached statistical significance (*p* < 0.001). Participants perceived the original version as the most positive, followed by the rhythm-only and melody-only versions, respectively. Moreover, participants perceived the original version as being the most relaxed, followed by the rhythm-only version and then the melody-only version (see Figure 2).

In addition, a significant group difference between music majors and nonmusic majors was found in their perception of the complexity of background music, indicating that music majors tended to perceive the background music as significantly simpler than nonmusic majors.

**Figure 3:**
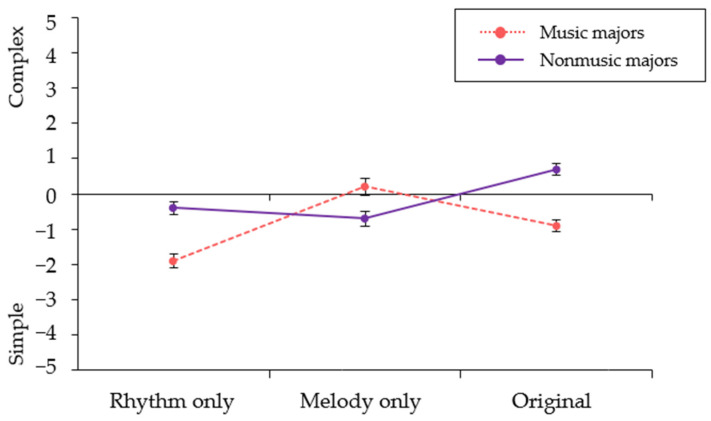
Perceived complexity of background music in music majors versus nonmusic majors. Error bars indicate standard errors.

The interaction effect between arrangement type and group was significant and such significant interaction was found for complexity. Music majors and nonmusic majors showed similar trends in perceiving emotional valence and arousal from background music depending on the arrangement type. However, music majors and nonmusic majors showed differences in their perception of the complexity of the presented background music. While music majors perceived the melody-only version as the most complex followed by the original version and then the rhythm-only version, nonmusic majors perceived the original version as the most complex and perceived the rhythm-only and melody-only versions to be simpler at a similar level (see Figure 3).

In this study, a multiple regression was calculated to predict cognitive performance (percentage of completed items and correct task performance) based on perceptions of background music (i.e., complexity, emotional arousal and valence; see Table 9). For model fitness, Durbin-Watson was between 1.5 and 2.0 and VIF was less than 10 for all calculations, supporting the hypothesis. For goodness-of-fit of the model for the percentage of completed items in each group, as measured by adjusted R^2^, the model explained 7.9% of the variance for music majors and 20.9% of the variance for nonmusic majors. For music majors, none of the perception measures contributed significantly to the model, while for nonmusic majors, only the perceived complexity significantly contributed to the model (B = −0.792, *p* = 0.043). Nonmusic majors’ percentage of completed items increased 0.8% of 1 point in the rating score, as the perceived complexity decreased.

For the prediction of correct performance for each group, the model explained 7.4% of the variance for music majors and 8.7% of the variance for nonmusic majors. The model significantly fits the model for cognitive performance (*p* < 0.001 for both groups). However, none of the perceived measures (i.e., complexity, arousal, and valence) significantly contributed to the model.

## 4. Discussion

This study examined how different arrangements of background music affected cognitive task performance between music majors and nonmusic majors. The target music was modified with pitch variation (i.e., melody-only version with isochronous rhythm) and rhythm variation (i.e., rhythm-only version with no pitch). While presented with the two arrangements and original version, the participants were asked to complete a task that required processing speed and sustained attention. Their cognitive performance (i.e., measured by the percentage of completed items in the test and percentage of accurately completed items) and perceptions on the background music were analyzed depending on the arrangement type.

First, when they were asked to rate whether the background music was interfering with, neutral to, or beneficial for cognitive performance, 60% of participants rated that the background music was interfering with their performance. The estimated mean for the rating was −1.3 (SD = 3.0), indicating that participants tended to perceive the music as slightly interfering. Level of perceived interference or benefit also differed depending on the arrangement type, with the original music perceived as being the least interfering and the melody-only version perceived as being the most interfering. Still, the proportion of participants who rated the original music as beneficial for cognitive performance was less than 50%.

These results contradict earlier studies that applied the same music (Mozart’s Piano Sonata K.448) and demonstrated improved performance in spatial reasoning tasks compared to the no background music condition [5,8]. It is important to note that the present study differed from previous research in multiple ways. For example, the target music was modified by extracting some musical elements from the original music, and the participants were asked to consciously attend to and rate how background music affected their performance. The focus of this study was on which factors explained the mediating effect of music on concurrent cognitive performance, and the results regarding the participants’ unfavorable perceptions of music indicate that background music can take cognitive resources away from the presented cognitive task. Furthermore, the level of resources or cognitive load attracted by music depends on several factors, including how the music is processed, and this relates to intramusical characteristics [34,35,36]. However, since no music condition was not counterbalanced and task performance during silence and background music conditions was not directly compared, enhanced or decreased performance during background music conditions could not be determined. Further studies are needed that directly compare the no music and background music conditions, which would corroborate the actual effects of background music on cognitive performance.

Analyses of cognitive performance depending on arrangement type demonstrated that the two groups (music and nonmusic majors) differed in how many items they completed depending on the arrangement type; however, group differences were not found in task performance accuracy. No significant differences in correct task performance were attributed to a ceiling effect, given that both groups demonstrated a 93% correct performance rate across the three arrangement types. Furthermore, nonmusic majors demonstrated a similar level of engagement across the arrangement types, but music majors demonstrated a decrease in task completion for the original music compared to the rhythm-only and melody-only versions. Given that decreased engagement in this type of task was found to relate to the information prioritization or inhibition of competing responses [24], the original music may shift music majors’ attention away from the target stimulus and make them more readily distracted by other stimuli, including music. This might be due to music majors being more familiar with the target music, which aligns with previous studies demonstrating that familiar music can attract attentional resources as a competing stimulus [17,18].

Further analyses of differences in implicit processing of external input (i.e., participants’ perceptions of background music) between groups provided a more detailed explanation. For perceptions on the background music, both factors of arrangement type and musical background affected cognitive performance, and music majors and nonmusic majors were influenced differently by the arrangement of background music. The groups perceived the emotional valence and arousal of each arrangement of background music differently but in a similar pattern; they perceived the melody-only version as the most arousing and the least positive and the original music as the most relaxing and the most positive. This can be explained by the interrelationship between perceived arousal and pleasantness (or aversion) that indicates increased arousal beyond an optimal level, which leads to aversion [37].

Pitch information may be a more salient cue than rhythm in recognizing the content of musical stimuli, because differences between basic elements (e.g., different pitch classes versus different sets of intervals between notes) are more distinctive [38,39]. In this study, the melody-only version, dominantly a pitch contour without a rhythmic component, might have seemed more deviant from the musical expectation and led to increases in arousal and decreases in emotional positivity. Still, clear-cut interpretation of the pitch’s effects on attentional load cannot be drawn. In this study, the music was arranged to differentiate specific intra-musical elements; however, the melody-only version still may have had a temporal (rhythmic) foundation. It may be possible that other variables (e.g., number of voices or timbre of the instruments) limit the investigation of the sole influence of pitch or rhythmic components. As such, additional studies are needed to systematically investigate these potentially relevant variables (e.g., isochronous beat with and without pitch, simple versus complex rhythm pattern with and without pitch).

Regarding perceived complexity, how the two groups perceived each arrangement type of background music differed. The music majors perceived the rhythm-only and original versions as having similar complexity, whereas the nonmusic group perceived the original music as the most complex, while perceiving the rhythm-only and melody-only versions as being simpler and at a similar level. These results indicate that the music majors tended to perceive increased information (i.e., increased complexity) with the changing aspects of music [10,17,19] that deviate from expectancy as in the melody-only version, whereas the nonmusic group tended to be influenced by the amount of information that constituted the music, such as the number of notes played at the same time (e.g., a single voice in the rhythm-only and melody-only versions, versus multiple voices in the original version). However, further investigation is needed using arrangements with different voices (i.e., a single melody line versus multiple voices such as chord progression as an accompaniment) to confirm which specific element contributes to the amount of information perceived as complex.

Furthermore, the results of multiple regression in this study demonstrated interesting findings. While music majors’ perceptions on background music did not significantly contribute to predicting their cognitive performance (either percentage of completed items or performance accuracy), the complexity of background music perceived by nonmusic majors significantly predicted their level of engagement in the cognitive task. The music majors’ results might be attributed to the fact that the variations found across the arrangement types were not differently perceived, given that the original music played in this study had structural regularity, and such structural features were maintained across the versions. Given that music majors tended to perceive the original music as simple and positive but that the percentage of their completed items decreased during this music condition, music majors’ cognitive performance with background music may be influenced by other factors (e.g., familiarity or preference) and not emotional arousal, mood or load of processing music information.

Meanwhile, nonmusic majors tended to increase their cognitive performance for music they perceived as simpler, and this perceived complexity of music significantly affected their cognitive performance. Given that additional attentional control would not be recruited when the concurrent stimulus (e.g., background music) is not competitive or conflicting with the target stimulus (e.g., cognitive task; [24,25]), this indicates that constituting elements might influence the level of demand on attentional control for nonmusic majors who might possess relatively fewer resources and require more effort to process music as information. Furthermore, the different results between music majors and nonmusic majors extend the potential role of background music from a mood-inducing or arousal-mediating agent to creating concurrent or competing information that would use attentional resources. Moreover, the level of cognitive resources that might be allocated to music (not to the target cognitive task) would depend on the resources that task performers or listeners already have [10,19]. Whether music can have a facilitatory effect on cognitive performance by inducing an optimal level of arousal or whether music may demand excessive control to the extent it disrupts, the effective attention strategy is a critical consideration when using background music during cognitive performance and such consideration necessarily involves individual factors, including perceptual differences.

## 5. Conclusions

In this study, a comparative analysis of perceptions of different musical arrangements indicated that music-mediating attentional sources or orientation can be an important factor for cognitive task performance. Extending previous attempts, this study demonstrated that the type of background music with varying intramusical elements and the way of constituting or modifying the elements should be considered. This current finding extends the role of background music from inducing optimally aroused states to mediating the expectancy and complexity that determines how background music affects the concurrent cognitive performance. The processing of music via concurrent attentional resources affects performance in terms of inducing an optimal level of arousal for such performance depending on attentional and cognitive demands. Given that a familiarity with music was not controlled in this study, further studies are recommended to investigate how familiarity influences listeners’ musical expectancies when musical elements are modified. Future studies should use unfamiliar music with different acoustical features and systematically investigate individuals’ prior experiences, perceptions, and ability to process music information as mediating factors related to cognitive performance.

## Figures and Tables

**Table 1:** Participants’ demographic information.

Variable	Total (*n* = 126)	Music Majors (*n* = 70)	Nonmusic Majors (*n* = 56)	*t*
Sex (Male:Female), *n*(%)	37 (29.4): 89 (70.6)	10 (14.3): 60 (85.7)	27 (48.2): 29 (51.8)	-
Age in years, M ± SD	22.6 ± 2.9	23.0 ± 3.1	22.0 ± 2.5	2.144 *
Years of music education/training, M ± SD	6.4 ± 6.7	10.9 ± 5.8	0.9 ± 1.1	14.209 ***
Duration of music listening per day, *n*(%)				
Never	4 (3.2)	1 (1.4)	3 (5.4)	2.610 ^1^
More than 1 min but less than 30 min	18 (14.3)	9 (12.9)	9 (16.1)	
30 min to 1 h	41 (32.5)	25 (35.7)	16 (28.6)	
More than 1 h to 2 h	32 (25.4)	29 (27.1)	13 (23.2)	
More than 2 h	31 (24.6)	16 (22.9)	15 (26.8)	
Listening habit ^2^, 1 to 5, M ± SD				
Only listening to music	2.9 ± 1.0	3.3 ± 1.2	2.6 ± 1.2	3.572 ***
Listening while performing other tasks	3.3 ± 1.2	3.3 ± 1.2	3.3 ± 1.2	0.434

^1^ Chi-square (χ^2^). ^2^ 5-point Likert scale with ratings from 1 (never) to 5 (always). * *p* < 0.05. *** *p* < 0.001.

**Table 2:** Features of each background arrangement.

Arrangement	Feature	Example (Measures 1–4)
Original	Consisted of combined rhythm, melody, and harmonic sequences in the original music	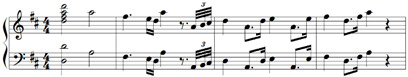
Melody-only	Consisted of the melodic contour of the top voice in the original version but with isochronous beats	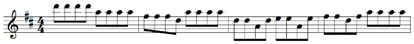
Rhythm-only	Consisted of the rhythm of the top melody in the original music with melodic, harmonic, and texture features omitted	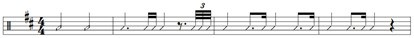

**Table 3:** Baseline measures of level of arousal and cognitive task performance.

Variable	Total (*N* = 126)	Music Majors (*n* = 70)	Nonmusic Majors (*n* = 56)	*t*	*p*
Level of perceived arousal (0–5)	2.9 ± 0.6	2.9 ± 0.6	2.9 ± 0.7	0.028	0.978
Percentage of completed items in silence	65.6 ± 16.4	65.6 ± 16.7	65.6 ± 16.1	0.017	0.987
Correct task performance in silence	93.5 ± 10.0	93.1 ± 10.6	93.9 ± 9.2	−0.447	0.655

**Table 4:** Perceived benefits of background music for each arrangement type.

Category	Total	Arrangement Type			χ^2^/*F*
		Rhythm-Only	Melody-Only	Original	
Number of respondents, *n* (%)					
Detrimental	228 (60.3)	71 (56.3)	98 (77.8)	59 (46.8)	29.412 ***
Neutral	48 (12.7)	22 (17.5)	8 (6.3)	18 (14.3)	
Beneficial	102 (27.0)	33 (26.2)	20 (15.9)	49 (38.9)	
Mean rating, M ± SD	−1.4 ± 3.0	−1.1 ± 2.8	−2.5 ± 2.8	−0.3 ± 2.9	20.454 ***

Note. The number of respondents depending on the arrangement type was analyzed using a chi-square test and the difference in mean rating was analyzed using a repeated measures ANOVA. *** *p* < 0.001.

**Table 5:** Level of performance of cognitive tasks and perceptions of background music depending on the musical arrangement for music majors and nonmusic majors.

Variable	Music Majors (*n* = 70)			Nonmusic Majors (*n* = 56)		
	Rhythm-Only	Melody-Only	Original	Rhythm-Only	Melody-Only	Original
Task performance						
Percentage of completed items	79.4 ± 15.2	80.2 ± 14.6	73.3 ± 16.6	82.0 ± 14.3	80.1 ± 16.9	79.1 ± 15.8
Correct task performance	93.7 ± 8.6	94.2 ± 7.6	93.3 ± 9.9	92.6 ± 9.4	93.2 ± 8.9	93.3 ± 9.6
Perception of music						
Complexity	−1.9 ± 3.0	0.2 ± 3.6	−0.9 ± 2.9	−0.4 ± 2.9	−0.7 ± 3.8	0.7 ± 2.4
Arousal	1.3 ± 2.5	2.8 ± 2.3	−0.6 ± 2.6	0.8 ± 2.2	3.2 ± 1.8	0.2 ± 2.7
Valence	0.5 ± 3.1	−2.1 ± 2.7	2.3 ± 2.5	1.5 ± 2.7	−2.2 ± 2.4	2.4 ± 2.5

**Table 6:** Comparison of cognitive task performance depending on musical arrangement and group.

Variable	*F*	*df*	*p*	*η* ^2^
Percentage of completed items				
Arrangement	1.659	2, 244	0.192	0.009
Group	3.319	1, 122	0.072	0.008
Arrangement × group	3.921	2, 244	0.021 *	0.061
Correct task performance				
Arrangement	5.326	2, 244	0.005 **	0.042
Group	2.186	1, 122	0.142	0.018
Arrangement × group	0.867	2, 244	0.422	0.007

* *p* < 0.05. ** *p* < 0.01.

**Table 7:** The results of two-way MANOVA with three measures of listeners’ perceptions of music.

Independent Variable	Two-Way MANOVA Results			
	Wilks’ λ	*F*	*p*	*η* ^2^
Main effects				
Arrangement	0.586	37.836	<0.001 ***	0.235
Group	0.974	3.336	0.020 *	0.026
Interaction effects				
Arrangement × Group	0.943	3.646	0.001 **	0.029

* *p* < 0.05. ** *p* < 0.01. *** *p* < 0.001.

**Table 8:** The results of tests of between-subjects’ effects.

**Independent Variable**	**Variable**	***F***	***d*** ***f***	***p***	***η* ^2^**
Arrangement	Complexity	3.984	2, 372	0.020 *	0.021
	Valence	97.137	2, 372	<0.001 ***	0.343
	Arousal	57.321	2, 372	<0.001 ***	0.236
Group	Complexity	5.254	1, 372	0.022 *	0.014
	Valence	1.490	1, 372	0.223	0.004
	Arousal	0.874	1, 372	0.351	0.004
Arrangement × Group	Complexity	6.158	2, 372	0.002 **	0.032
	Valence	1.384	2, 372	0.252	0.007
	Arousal	2.360	2, 372	0.096	0.013

* *p* < 0.05. ** *p* < 0.01. *** *p* < 0.001.

**Table 9:** Regression coefficients for predicting cognitive performance based on perceptions of music in music majors and nonmusic majors.

**Variable**	**Music Majors (*n* = 70)**							
	**Percentage of Completed Items**				**Correct Task Performance**			
	**B**	**Beta ** ** (*β*) **	***T***	***p***	**B**	**Beta ** ** ( *β*) **	***t***	***p***
Complexity	−0.113	−0.024	−0.318	0.750	−0.167	−0.063	1.250	0.213
Arousal	0.343	0.072	0.816	0.415	−0.237	−0.090	−1.498	0.136
Valence	0.383	0.069	0.787	0.432	−0.338	−0.110	−1.843	0.067
**Variable**	**Nonmusic Majors (*n* = 56)**							
	**Percentage of Completed Items**				**Correct Task Performance**			
	**B**	**Beta ** ** (*β*) **	***T***	***p***	**B**	**Beta ** ** (*β*) **	***t***	***p***
Complexity	−0.792	−0.158	−2.041	0.043 *	0.068	0.023	0.580	0.563
Arousal	−0.363	−0.075	−0.833	0.406	0.108	0.037	0.812	0.418
Valence	−0.422	−0.071	−0.781	0.436	0.057	0.016	0.347	0.729

* *p* < 0.05.
